# Anti-inflammatory effects of metformin in gestational diabetes: Systematic review and meta-analysis of randomized trials and preclinical animal pregnancy models study protocol

**DOI:** 10.1371/journal.pone.0361047

**Published:** 2026-11-12

**Authors:** Israel Ogwuche Ogra, Aghogho Oyibo, Fabrice Banadzem Kernyuy, Ayomide Oluwaseun Adereti, Babalola Ooreofe Feranmi, Kayode Olayinka Afolabi, Glory Chinuru Musa, Vivian Onyinye Ojiakor, Umar-farouq A. Mahe, Oluwatoyin Osinimega Ojo, Mahmoud Werfalli, Samuel Ohiomokhare, Kodjovi Sossou, Olayinka Sunday Okoh, Ezekiel Musa

**Affiliations:** 1 UNESCO International Centre for Biotechnology, Nsukka, Enugu State, Nigeria; 2 Biological Institute, Tomsk State University, Tomsk, Russia; 3 Department of Biochemistry, Christland University, Abeokuta, Nigeria; 4 Drosophila Research and Training Centre, Ibadan, Nigeria; 5 Department of Biochemistry, Covenant University, Ota, Ogun State, Nigeria; 6 Faculty of Veterinary Medicine, University of Nigeria, Nsukka, Nigeria; 7 Department of Chemical Sciences, Bamidele Olumilua University of Education, Science and Technology, Ikere, Ekiti, Nigeria; 8 Centre for Global Health, Anchor University, Ayobo, Lagos, Nigeria; 9 Molecular Epidemiology and Public Health Research Group (MEPHREG), Department of Biological Sciences, Anchor University, Ayobo, Lagos, Nigeria; 10 Independent Researcher; 11 Department of Anatomy, Faculty of Basic Medical Sciences, Enugu State University of Science and Technology Enugu, Enugu, Enugu State, Nigeria; 12 Department of Human Anatomy, Northwest University, Kano, Kano State, Nigeria; 13 Department of Chemical Sciences, Faculty of Natural and Applied Sciences, Anchor University, Ayobo, Lagos State, Nigeria; 14 Department of Family and Community Medicine, Faculty of Medicine, University of Benghazi, Benghazi, Libya; 15 Laboratory of Biomedical, Food and Environmental Health Sciences – Biomedical Sciences and Bioactive Substances Research Unit (LaSBASE – UR–2SB), Ecole Supérieure des Techniques Biologiques et Alimentaires (ESTBA), University of Lomé, Lomé, Togo; 16 North Cumbria Integrated Care NHS Foundation Trust, Carlisle, United Kingdom; 17 Department of Internal Medicine, Kaduna State University, Kaduna, Nigeria; UFRN: Universidade Federal do Rio Grande do Norte, BRAZIL

## Abstract

**Background:**

Gestational diabetes mellitus (GDM) is a significant complication that can occur during pregnancy. It is characterized by glucose intolerance that first arises during pregnancy and is associated with insulin resistance, which is linked to elevated inflammatory markers. Metformin has emerged as a promising treatment option for GDM, with growing evidence supporting its anti-inflammatory effects. These effects are thought to be mediated by activation of AMP-activated protein kinase (AMPK) and inhibition of NF-κB signaling. This systematic review aims to assess the anti-inflammatory effects of metformin in GDM, considering both clinical and preclinical studies.

**Methods:**

A systematic review of all randomized controlled trials and controlled *in vivo* experiments for animals published until 2026 will be carried out. A comprehensive search of all published articles indexed in PubMed/MEDLINE, the Cochrane Library, Scopus, CINAHL (EBSCOhost), Embase, and Web of Science will be performed. Studies will be screened for eligibility by title, abstract, and full text in duplicate by two independent reviewers. For data where meta-analysis is not possible, a narrative analysis will be conducted using themes derived from the data. For data that allow meta-analysis, a random-effects meta-analysis will be conducted. This systematic review will be reported in accordance with the Preferred Reporting Items for Systematic Reviews and Meta-Analysis (PRISMA).

**Discussion:**

The study will utilize meta-analysis to establish the anti-inflammatory effects of metformin in GDM, potentially influencing clinical practice and guidelines. Additionally, validating metformin’s anti-inflammatory effects has important implications beyond immediate management of GDM, extending to long-term adverse outcomes for both the mother and the offspring.

**Systematic review registration:**

**PROSPERO** CRD420261337881

## Introduction

Gestational diabetes mellitus (GDM) is a notable complication during pregnancy. It is defined as glucose intolerance first identified in pregnancy, which eventually becomes evident in the second or third trimester [1,2]. In addition to its immediate effects, GDM largely contributes to the global diabetes burden. It predisposes both the mother and their children to an increased risk of developing type 2 diabetes later in life [1,2]. GDM is globally prevalent, and risk factors, such as advanced maternal age, obesity, family history of diabetes, and prior GDM, play influential roles [3–5]. Recent evidence from Sub-Saharan Africa observed that obesity and older maternal age are major predictors of GDM [3]. Similarly, a study from Bangladesh reported a high prevalence of GDM and its strong association with adverse outcomes for both mothers and infants [5]. The persistent rise in GDM prevalence underscores growing concerns about its long-term consequences, such as progression to type 2 diabetes.

GDM is commonly accompanied by insulin resistance, which is closely connected with higher levels of inflammatory markers, such as interleukin-6 (IL-6), tumor necrosis factor-alpha (TNF-α), C-reactive protein (CRP), and serum amyloid A (SAA) [6,7]. This elevated inflammatory condition is associated with several negative outcomes, including preeclampsia, fetal macrosomia, and a greater chance of cesarean delivery [8–10]. IL-6 and TNF-α play a key role in systemic inflammation that aggravates insulin resistance, resulting in a cycle of intensified hyperglycaemia [11]. Notably, TNF-α disrupts insulin receptor signalling and reduces insulin sensitivity, thereby contributing to glucose metabolism impairments observed in pregnant women [12,13]. Similarly, higher TNF-α concentrations have been positively associated with increased insulin resistance, as shown by homeostasis model assessment (HOMA) indices in women diagnosed with GDM [14]. The search for a preventive and therapeutic regimen for GDM with anti-inflammatory properties has led to the use of several therapeutic options, such as metformin, to manage GDM.

Metformin is gaining attention as a valuable treatment option for GDM, largely because of its oral availability, favorable safety profile, and potential to improve outcomes for both mother and baby [15,16]. Although metformin was initially introduced as the first-line therapy for type 2 diabetes, it is increasingly adopted in the management of GDM due to strong evidence of its effectiveness [15,16]. Its major strength lies in its ability to control blood glucose levels [17]. Metformin can reduce overall insulin requirements by 20%–70%, though it does not entirely replace insulin therapy [18]. Another important consideration is its safety during pregnancy [19–21]. Research has shown that while metformin is excreted in breast milk, no adverse effects have been reported in breastfed infants [19]. Beyond pregnancy, the drug also plays a preventive role [22]. Findings from the Diabetes Prevention Programme revealed that metformin significantly reduces the risk of type 2 diabetes in women with a history of GDM [22].

Interestingly, emerging evidence indicates that metformin may also exert anti-inflammatory effects through mechanisms such as activating AMP-activated protein kinase (AMPK) and inhibiting nuclear factor kappa-light-chain-enhancer of activated B cells (NF-κB) signalling [23–25]. AMPK activation by metformin has been shown to suppress TNF-α-driven inflammation, a key factor in insulin resistance and obesity-related inflammation [26]. The NF-κB pathway is often overactive in obesity and type 2 diabetes, both of which are closely tied to GDM, and it is a central modulator of inflammation. Evidence suggests that metformin reduces this activity, helping to control inflammation. For instance, Zhou et al. [27] found that metformin lowers nuclear NF-κB expression, while Kim et al. [28] demonstrated that it inhibits NF-κB activation in macrophages through AMPK-dependent pathways when exposed to inflammatory stimuli such as lipopolysaccharide (LPS). These findings collectively highlight metformin’s promising role as both a glucose-lowering and anti-inflammatory therapy in the management of GDM.

Despite the growing research suggesting anti-inflammatory properties of metformin in gestational diabetes, comprehensive evaluations of these effects in both clinical and preclinical contexts have not been systematically aggregated. The lack of a systematic understanding of metformin’s anti-inflammatory mechanisms in GDM limits progress toward optimizing therapeutic strategies. Therefore, this systematic review aims to evaluate the anti-inflammatory effects of metformin in gestational diabetes in both clinical and preclinical contexts. A systematic review and meta-analysis that collates data from randomized trials and preclinical animal models of pregnancy can provide clarity on metformin's role in modulating inflammatory processes in GDM, potentially influencing clinical practice and guidelines. Additionally, validating metformin’s anti-inflammatory effects has important implications beyond immediate management of GDM, extending to long-term adverse outcomes for both the mother and the offspring.

### Aim

The systematic review and meta-analysis aim to synthesise the current literature on the anti-inflammatory effects of metformin in GDM, both from clinical studies and preclinical animal pregnancy models.

**The specific objectives are:**

1. To determine the concentrations of inflammatory markers in GDM patients treated with or without metformin.
2. To evaluate the outcomes of pregnancy in metformin-treated GDM and other therapies

## Materials and methods

### Design

The protocol was in line with the Preferred Reporting Items for Systematic Reviews and Meta-Analysis Protocol (PRISMA-P) guidelines (S1 File). This study will synthesize literature on GDM, including all randomized controlled trials and controlled in vivo animal experiments published until 2026. The population will be pregnant women diagnosed with GDM and pregnant animal models. The intervention will be metformin administration during pregnancy. At the same time, the comparator will be either a placebo, no treatment, insulin, other antihyperglycemic agents, or vehicle-treated controls (in animal studies). A meta-analysis will be conducted when sufficient data with low heterogeneity are available. The data retrieval will start in September 2026; screening of records will be finalized by November 2026, while data extraction will be completed by December 2026, and results should be available by March 2027.

The literature search will be restricted to studies published in English because the review team has sufficient proficiency to accurately assess, extract, and critically appraise English-language publications. This restriction will facilitate consistent interpretation of methodological details, intervention characteristics, outcome measurements, and risk-of-bias information while ensuring accurate data extraction across the included studies. In addition, translation of non-English articles may introduce inconsistencies or errors in the interpretation of study methods and results and may not be feasible within the resources and timeframe of the review. Nevertheless, the potential for language bias associated with this restriction will be acknowledged as a limitation of the review, and the findings will be interpreted accordingly.

### Settings

Hospital- and laboratory-based studies

#### Inclusion criteria.

Pregnant women diagnosed with GDM and pregnant animals (rodents, rabbits, and other mammalian models) used as pregnancy models; studies modelling hyperglycaemia/GDM will be included, and studies of healthy pregnant animals are also eligible if metformin is given during pregnancy. Healthy pregnant animal models will be eligible for inclusion as a prespecified complementary evidence category alongside experimental models of gestational diabetes mellitus (GDM). Their inclusion is justified by the objective of characterizing the anti-inflammatory effects of metformin during pregnancy and distinguishing effects attributable to metformin itself from those occurring specifically in the context of GDM-associated metabolic and inflammatory dysfunction. Healthy pregnant and GDM animal models will not be considered interchangeable populations for the primary quantitative synthesis. Animal studies involving GDM models will constitute the primary population for estimating the effect of metformin on inflammatory outcomes associated with GDM, whereas healthy pregnant models will be synthesized separately to characterize metformin-associated inflammatory effects under physiological pregnancy conditions. All RCTs for analysis will have measured at least one of the following inflammatory markers in blood samples: CRP, adiponectin, TNF-α, IL-1β, resistin, and IL-6. Diagnosis of GDM will be defined according to the WHO 1999/2013, the American Diabetes Association (ADA), and the IADPSG diagnostic definitions and criteria [29–33].

#### Exclusion criteria.

In vitro and ex vivo studies, case reports, reviews, commentaries, editorials, letters, and studies without primary data or explicit description of methods, or both, will be excluded. Studies on non-pregnant humans and animals will be excluded.

### Definition of outcomes

i) Preeclampsia: It is defined as hypertension after 20 weeks of gestation with proteinuria or end-organ dysfunction. Inflammation and endothelial dysfunction increase the risk of preeclampsia [34].
ii) Preterm Birth (<37 weeks): It is defined as delivery before 37 completed weeks of gestation. It is linked to maternal systemic and placental inflammation [35].
iii) Macrosomia/Large-for-Gestational-Age (LGA): It is defined as birth weight ≥4000 g or >90th percentile for gestational age. Inflammation and maternal hyperglycemia contribute to fetal overgrowth [36].

### Patient and public involvement

No participant recruitment will be needed for the study.

#### Search strategy for identification of relevant studies.

A sensitive search strategy will be used to identify and retrieve articles published in English and indexed in PubMed/MEDLINE, Embase, the Cochrane Library, CINAHL (EBSCOhost), Scopus, and Web of Science. A search strategy using Medical Subject Headings and free text will be utilized to retrieve articles published in these databases until 2026 to capture all relevant studies (S1 Table). Unpublished literature will be sought from experts in the field. In contrast, grey literature, such as reports, will also be reviewed for relevant information from websites like BIOSIS, CAMARADES, SYRCLE registry, WHO, Google Scholar, and Clinical Trials Registry. The support of an experienced librarian will be solicited for validation and cross-examination of the search strategy.

### Study selection

Two reviewers (IOO and AO) will independently screen article titles and abstracts to identify eligible studies. Any discrepancies and doubts will be addressed through deliberation and consultation between the two reviewers. Disagreements will be resolved through discussion and, when necessary, through mutual resolution with a third and more experienced reviewer in the field (EM). The full texts of eligible articles will be retrieved and analyzed by the two reviewers (IOO and AO). Where necessary, further information will be sought from the study authors. Reasons for excluding articles will be documented. The entire study selection process will be described (Fig 1).

**Fig 1:**
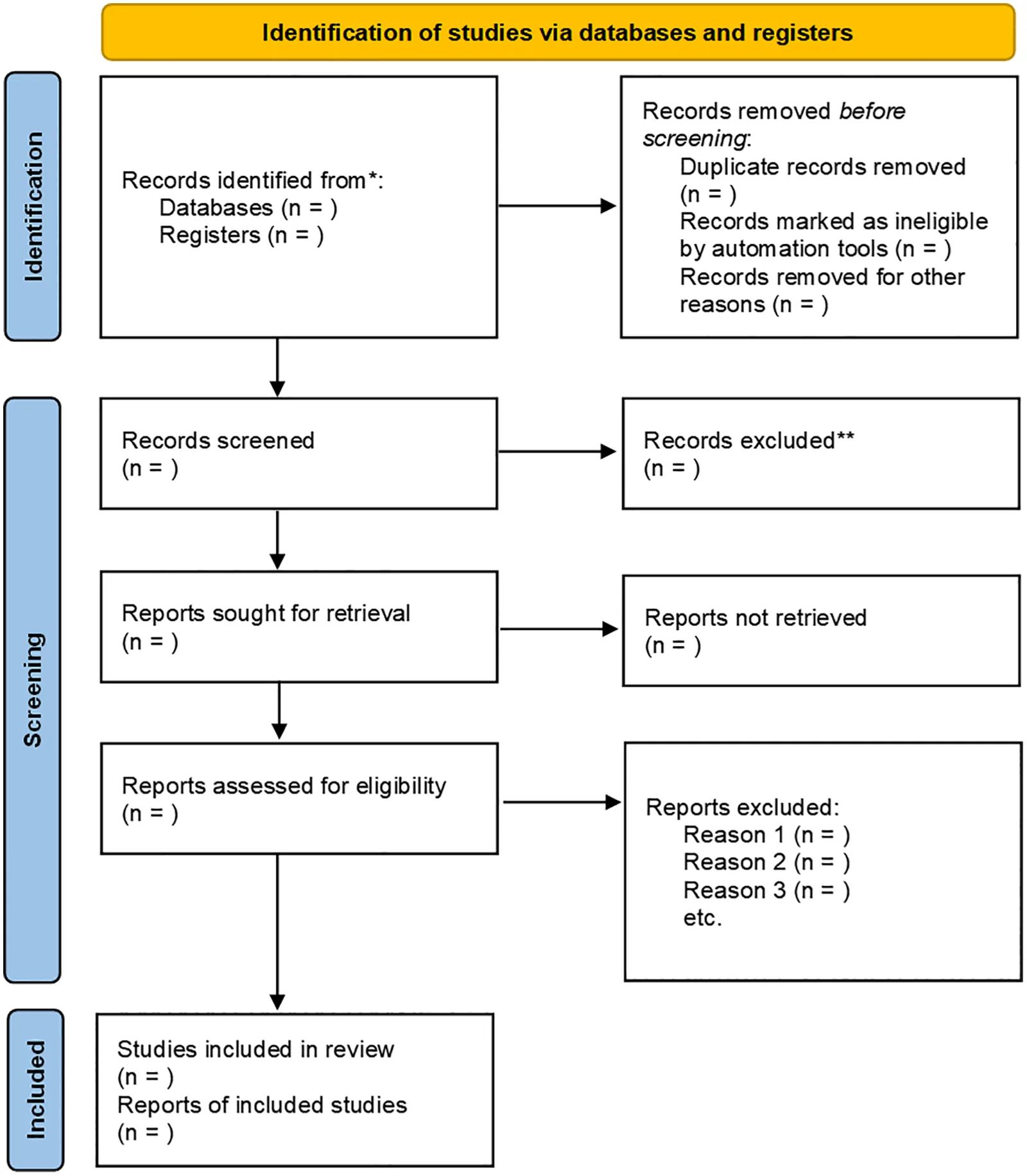
PRISMA 2020 flow diagram for new systematic reviews, which included searches of databases and registers only. *Consider, if feasible to do so, reporting the number of records identified from each database or register searched (rather than the total number across all databases/registers). **If automation tools were used, indicate how many records were excluded by a human and how many were excluded by automation tools.

### Data extraction

The Burden of Disease (BOD) Review Manager, developed by the South African Medical Research Council, will be used by two independent reviewers [37] to extract and record research data. The data to be extracted include general study information, author, year of publication, title of study, study design, country/region, period, journal, and objective of the study. For the study population, sample size (total, intervention, control), maternal age, gestational age at enrollment/intervention, pre-pregnancy BMI/obesity status, diagnostic criteria for GDM (IADPSG, WHO, ADA, etc.), inclusion and exclusion criteria, and baseline comorbidities (hypertension, PCOS, etc.) will be recorded for humans. For animal models, the following will be recorded: animal species/type, strain, method of GDM induction, gestational day of intervention, and pregnancy outcomes. Data on the intervention, such as dose, route of administration, duration of treatment, timing of initiation (preconception, early pregnancy, mid-pregnancy, late pregnancy), and compliance/adherence (reported in human RCTs), will be extracted.

Similarly, data on comparators such as placebo, insulin therapy, diet, lifestyle modification, and no treatment (in animal models) will be recorded. Primary outcome data, such as CRP / hs-CRP, IL-6, TNF-α, other cytokines (IL-1β, IL-10, MCP-1, adiponectin, leptin) will be recorded. Likewise, secondary maternal outcomes, glycemic outcomes (fasting glucose, OGTT values, HbA1c), insulin resistance indices, gestational weight gain, preeclampsia incidence, mode of delivery, and preterm birth (<37 weeks) will be noted. Secondary fetal/neonatal outcomes like birth weight, macrosomia incidence, neonatal hypoglycemia, NICU admission, respiratory distress, and perinatal mortality. Besides, long-term outcomes such as maternal postpartum progression to type 2 diabetes and offspring outcomes will be noted if reported.

### Risk of bias assessment

Risk of bias and study quality will be independently assessed by two reviewers (IOO and AO) for clinical RCTs using the Cochrane Risk of Bias 2 (RoB 2) tool. Whereas, for the animal studies, the SYRCLE risk-of-bias tool will be utilized. External and internal validity will be assessed by resolving differences among reviewers over the risk of bias through consensus or by involving an additional reviewer when necessary.

### Data synthesis and heterogeneity assessment

Crude numerators and denominators from each included study will be used to recalculate study-specific effects. Meta-analysis will be conducted when at least two independent studies provide sufficiently comparable quantitative data for the same population, intervention, comparator, outcome, and assessment time point. Estimates of metformin’s anti-inflammatory impact will be synthesised separately for clinical and preclinical studies. Studies will be pooled only when clinical, methodological, and experimental characteristics are sufficiently similar to justify quantitative synthesis. A meta-analysis will be conducted for variables comparable across studies, using a random-effects model to generate pooled effect estimates. Statistical heterogeneity will be assessed with Cochrane’s Q test and quantified using the I² statistic [38]. Planned subgroup analyses include outcome type, maternal BMI categories, timing of metformin initiation, assay method or sample source, animal species or model, and comparator type. Publication bias will be evaluated if an adequate number of studies are available using funnel plots and Egger’s regression test, with p < 0.10 considered indicative of bias [39]. Inter-rater reliability during study selection and risk-of-bias assessment will be quantified using Cohen’s κ statistic [40].

Statistical analyses will be performed using R statistical software with the *metafor* package. The *metafor* package was selected because it provides a comprehensive framework for meta-analysis and supports the calculation of effect sizes for both continuous and dichotomous outcomes, as well as fixed- and random-effects models, subgroup analysis, meta-regression, and graphical assessment of meta-analytic results [41]. For dichotomous outcomes, risk ratios (RRs), odds ratios (ORs), or risk differences (RDs) will be calculated from 2 × 2 contingency tables, as appropriate. For continuous outcomes, mean differences (MDs) will be used when outcomes are measured using the same scale and standardized mean differences (SMDs) when conceptually similar outcomes are assessed using different measurement scales. The selection of effect measures will follow recommendations of the Cochrane Handbook for Systematic Reviews of Interventions [42]. Where statistical analysis is not feasible, the findings will be summarized qualitatively by grouping results into themes, patterns, or categories, highlighting consistencies, differences, and trends across studies, and discussing the strengths, quality, and limitations of the evidence.

### Confidence in cumulative evidence

The quality of evidence will be rated using the GRADE framework [43–45]. This framework evaluates key domains, including study design, risk of bias, inconsistency, indirectness, imprecision, and publication bias. Based on these domains, the certainty of the evidence will be rated as “high”, “moderate”, “low”, or “very low.” [43].

### Review reporting

This systematic review will be reported in accordance with the updated Preferred Reporting Items for Systematic Reviews and Meta-Analysis (PRISMA) [46].

### Ethics and dissemination

This study will not involve data collection from human participants, so it will not require ethical approval. We will publish our findings in a peer-reviewed journal and may also present them at conferences.

## Supporting information

S1 File PRISMA-P 2015 checklist. (PDF)

S1 Table Proposed search strategy. (PDF)
