# Supplementary material for: Anti-inflammatory effects of metformin in gestational diabetes: Systematic review and meta-analysis of randomized trials and preclinical animal pregnancy models study protocol
Source: PLoS One. 2026 Nov 12;21(11):e0361047. doi: 10.1371/journal.pone.0361047 (PMC13759117; doi:10.1371/journal.pone.0361047)
Supplement: Supplementary file 2 [file pone.0361047.s002.pdf]

S1 Table Proposed Search Strategy

**Database/Category**	**Search Strategy**
**PubMed (clinical)**	(metformin[Mesh] OR metformin[tiab] OR "metformin hydrochloride"[tiab])<br><br>AND ("gestational diabetes"[Mesh] OR "gestational diabetes"[tiab] OR "pregnancy diabetes"[tiab] OR (pregnancy[tiab] AND diabetes[tiab]))<br><br>AND (inflamm*[tiab] OR CRP[tiab] OR "C-reactive protein"[tiab] OR IL-6[tiab] OR "interleukin 6"[tiab] OR TNF[tiab] OR "tumor necrosis factor"[tiab] OR "tumour necrosis factor"[tiab] OR cytokine*[tiab])
**PubMed (animal)**	(metformin[Mesh] OR metformin[tiab] OR "metformin hydrochloride"[tiab])<br><br>AND (pregnan*[tiab] OR gestation[tiab] OR pregnant[tiab] OR gestational[tiab])<br><br>AND (rat[tiab] OR mouse[tiab] OR murine[tiab] OR rabbit[tiab] OR rodent*[tiab] OR animal*[tiab] OR model[tiab])<br><br>AND (inflamm*[tiab] OR CRP[tiab] OR IL-6[tiab] OR TNF[tiab] OR cytokine*[tiab])

**Embase (clinical)**	(metformin/exp OR metformin:ti,ab OR 'metformin hydrochloride':ti,ab)<br><br>AND ('gestational diabetes'/exp OR 'gestational diabetes':ti,ab OR 'pregnancy diabetes':ti,ab OR (pregnancy:ti,ab AND diabetes:ti,ab))<br><br>AND (inflamm*:ti,ab OR CRP:ti,ab OR 'C-reactive protein':ti,ab OR IL-6:ti,ab OR 'interleukin 6':ti,ab OR TNF:ti,ab OR 'tumor necrosis factor':ti,ab OR 'tumour necrosis factor':ti,ab OR cytokine*:ti,ab)
**Embase (animal)**	(metformin/exp OR metformin:ti,ab OR 'metformin hydrochloride':ti,ab)<br><br>AND (pregnan*:ti,ab OR gestation:ti,ab OR pregnant:ti,ab OR gestational:ti,ab)<br><br>AND (rat:ti,ab OR mouse:ti,ab OR murine:ti,ab OR rabbit:ti,ab OR rodent*:ti,ab OR animal*:ti,ab OR model:ti,ab)<br><br>AND (inflamm*:ti,ab OR CRP:ti,ab OR IL-6:ti,ab OR TNF:ti,ab OR cytokine*:ti,ab)
**Scopus (clinical)**	(metformin OR metformin OR "metformin hydrochloride")

	AND ("gestational diabetes" OR "gestational diabetes" OR "pregnancy diabetes" OR (pregnancy AND diabetes))<br><br>AND (inflamm* OR CRP OR "C-reactive protein" OR IL-6 OR "interleukin 6" OR TNF OR "tumor necrosis factor" OR "tumour necrosis factor" OR cytokine*)
**Scopus (animal)**	(metformin OR metformin OR "metformin hydrochloride")<br><br>AND (pregnan* OR gestation OR pregnant OR gestational)<br><br>AND (rat OR mouse OR murine OR rabbit OR rodent* OR animal* OR model)<br><br>AND (inflamm* OR CRP OR IL-6 OR TNF OR cytokine*)
**Web of Science (clinical)**	(ALL=metformin OR (TI=metformin OR AB=metformin) OR (TI="metformin hydrochloride" OR AB="metformin hydrochloride"))<br><br>AND (ALL="gestational diabetes" OR (TI="gestational diabetes" OR AB="gestational diabetes") OR (TI="pregnancy diabetes" OR AB="pregnancy

	diabetes") OR ((TI=pregnancy OR AB=pregnancy) AND (TI=diabetes OR AB=diabetes)))<br><br>AND ((TI=inflamm* OR AB=inflamm*) OR (TI=CRP OR AB=CRP) OR (TI="C-reactive protein" OR AB="C-reactive protein") OR (TI=IL-6 OR AB=IL-6) OR (TI="interleukin 6" OR AB="interleukin 6") OR (TI=TNF OR AB=TNF) OR (TI="tumor necrosis factor" OR AB="tumor necrosis factor") OR (TI="tumour necrosis factor" OR AB="tumour necrosis factor") OR (TI=cytokine* OR AB=cytokine*))
**Web of Science (animal)**	(ALL=metformin OR (TI=metformin OR AB=metformin) OR (TI="metformin hydrochloride" OR AB="metformin hydrochloride"))<br><br>AND ((TI=pregnan* OR AB=pregnan*) OR (TI=gestation OR AB=gestation) OR (TI=pregnant OR AB=pregnant) OR (TI=gestational OR AB=gestational))<br><br>AND ((TI=rat OR AB=rat) OR (TI=mouse OR AB=mouse) OR (TI=murine OR AB=murine) OR (TI=rabbit OR AB=rabbit) OR

	(TI=rodent* OR AB=rodent*) OR (TI=animal* OR AB=animal*) OR (TI=model OR AB=model))<br><br>AND ((TI=inflamm* OR AB=inflamm*) OR (TI=CRP OR AB=CRP) OR (TI=IL-6 OR AB=IL-6) OR (TI=TNF OR AB=TNF) OR (TI=cytokine* OR AB=cytokine*))
